# Massively parallel sequencing of mitochondrial genome in primary open angle glaucoma identifies somatically acquired mitochondrial mutations in ocular tissue

**DOI:** 10.1038/s41598-024-72684-6

**Published:** 2024-11-01

**Authors:** Neeru Amrita Vallabh, Brian Lane, David Simpson, Marc Fuchs, Anshoo Choudhary, David Criddle, Robert Cheeseman, Colin Willoughby

**Affiliations:** 1 Department of Eye and Vision Science, Institute of Life Course and Medical Sciences, University of Liverpool, Liverpool, L69 3BX UK; 2 St. Paul’s Eye Unit, Royal Liverpool University Hospital, Liverpool, L7 8XP UK; 3 Translational Radiobiology Group, Division of Cancer Sciences, University of Manchester, Manchester Academic Health Science Centre, Christie NHS Foundation Trust Hospital, Manchester, M20 4BX UK; 4 Wellcome-Wolfson Institute for Experimental Medicine, Queen’s University Belfast, Belfast, BT9 7BL UK; 5 Institute of Systems, Molecular and Integrative Biology, Biosciences Building, University of Liverpool, Liverpool, L69 7BE UK; 6 Genomic Medicine, Biomedical Sciences Research Institute, Ulster University, Coleraine, BT52 1SA UK

**Keywords:** Tenon’s fibroblasts, Glaucoma, Mitochondrial genome, Somatic mutations, Massively parallel sequencing, Mitochondrial DNA, Ocular hypertension, Optic nerve diseases, Genetics, Clinical genetics

## Abstract

Glaucoma is a sight threatening neurodegenerative condition of the optic nerve head associated with ageing and marked by the loss of retinal ganglion cells. Mitochondrial dysfunction plays a crucial role in the pathogenesis of neurodegeneration in the most prevalent type of glaucoma: primary open angle glaucoma (POAG). All previous mitochondrial genome sequencing studies in POAG analyzed mitochondrial DNA (mtDNA) isolated from peripheral blood leukocytes and have not evaluated cells derived from ocular tissue, which better represent the glaucomatous disease context. In this study, we evaluated mitochondrial genome variation and heteroplasmy using massively parallel sequencing of mtDNA in a cohort of patients with POAG, and in a subset assess the role of somatic mitochondrial genome mutations in disease pathogenesis using paired samples of peripheral blood leukocytes and ocular tissue (Tenon’s ocular fibroblasts). An enrichment of potentially pathogenic nonsynonymous mtDNA variants was identified in Tenon’s ocular fibroblasts from participants with POAG. The absence of oxidative DNA damage and predominance of transition variants support the concept that errors in mtDNA replication represent the predominant mutation mechanism in Tenon’s ocular fibroblasts from patients with POAG. Pathogenic somatic mitochondrial genome mutations were observed in people with POAG. This supports the role of somatic mitochondrial genome variants in the etiology of glaucoma.

**Supplementary Information:**

The online version contains supplementary material available at 10.1038/s41598-024-72684-6.

## Introduction

Glaucoma is an age-related neurodegenerative disease of the optic nerve head characterized by the death of retinal ganglion cells (RGCs), resulting in progressive visual field loss and subsequent blindness^1,2^. Primary open angle glaucoma (POAG) is the predominant subtype of glaucoma with the number of affected individuals estimated to be 111.8 million by 2040 because of increased life expectancy and an ageing population^3^. The pathogenesis of POAG is multifactorial and represents an interplay between genetic, vascular, mechanical, immunological and metabolic factors^1^. Despite this complexity, lowering intra-ocular pressure (IOP) is the only modifiable risk factor^4^. Even when IOP is adequately controlled, a significant number of people with POAG show progressive loss of visual field and ultimately blindness^5–8^. This has driven efforts to identify IOP-independent neuroprotective therapies for POAG^9,10^. Mitochondrial dysfunction plays a crucial role in the pathogenesis of neurodegeneration in POAG^9,11–14^. In fact, the inherent susceptibility of RGCs to mitochondrial dysfunction is demonstrated by inherited optic neuropathies^15^.

Leber’s hereditary optic neuropathy (LHON) and autosomal dominant optic atrophy (ADOA) are inherited mitochondrial optic neuropathies that are characterized by the selective neurodegeneration of RGCs^16^. In over 95% of cases, LHON results from maternally inherited single nucleotide mutations in mitochondrial DNA (mtDNA) encoding different mitochondrial protein subunits of respiratory complex I within the electron transport chain (ETC)^17^. In LHON, mtDNA defects are ubiquitously expressed but phenotypically limited to RGC neurodegeneration and visual loss highlighting the exquisite susceptibility of RGCs to mitochondrial dysfunction^9,13,16–18^. The phenotypic similarities of inherited optic neuropathies and glaucoma have prompted evaluation of the role of mitochondria in the pathogenesis of POAG^9,11,12,19,20^ and catalyzed clinical interest in the use of nicotinamide to provide metabolic support to RGCs^21–24^.

Mitochondrial oxidative phosphorylation (OXPHOS) complex-I impairment has been reported in peripheral blood lymphoblasts from LHON and POAG resulting in decreased mitochondrial respiration and ATP production^25,26^. Our group recently reported impaired mitochondrial cellular bioenergetics in glaucomatous ocular fibroblasts including basal respiration, maximal respiration and spare capacity^27^. Several groups, including ours, have investigated mtDNA defects in the pathogenesis of POAG^20,28–34^. The mitochondrial genome is organized as a circular, double-stranded DNA molecule in the mitochondrion and codes for only 37 genes across ~ 16,600 base pairs^35^. The mitochondrial genome can replicate independently of nuclear DNA, and in humans there are usually 100–10,000 separate copies of mtDNA present in each cell^36^. Homoplasmy describes the state when all copies of mtDNA are mutant and heteroplasmy is the state when only a proportion of the mtDNA is mutant^36^. Therefore, the coexistence of multiple mtDNA species (wild type mtDNA and mutated mtDNA variants) in a single cell or among cells within an individual makes mitochondrial diseases more complex and heterogeneous. The development of massively parallel or next generation sequencing technologies allows for accurate evaluation of these multiple mitochondrial genome variants, including the level of heteroplasmy, for each mtDNA variant^28–30,33,37^.

The clinical manifestations of a mitochondrial disease may only occur when the degree of mutated mtDNA exceeds a certain bioenergetic threshold, hence resulting in phenotypic variation, which in turn is dependent on the ratio of mutant to wild type cellular mtDNA (level of heteroplasmy)^12,38^. The bioenergetic threshold is usually at least 70–80% pathogenic mutant DNA to result in threshold effect^39^ but the threshold does vary between organs. This is dependent upon their energy requirements and reflects the inability of the remaining wild type mtDNA to compensate for the mutated mtDNA^38,40^. The bioenergetic threshold is cell and tissue specific and explains why the phenotype of LHON mtDNA defects are limited to RGC neurodegeneration^13^. Analysis of human leukocytes and skin fibroblasts has previously been used to characterize mtDNA mutations and dysfunction in glaucoma and other neurodegenerative diseases^25,26,41,42^. Cells derived from ocular tissues better represent the glaucomatous disease context and post-mortem studies have identified mitochondrial defects in the glaucomatous retina^43^ and lamina cribrosa cells cultured from the optic nerve head^44^. Mitochondrial dysfunction has also been studied in glaucoma using Tenon’s ocular fibroblasts^27,45^ and represent an alternative to post-mortem studies which are expensive, may lack detailed clinical data and limited in terms of sample size. Tenon’s ocular fibroblasts can be easily harvested at the time of cataract surgery and used as an assessable ocular cell type^27,46–48^.

All previous mitochondrial genome sequencing studies in POAG, were performed on mtDNA isolated from peripheral blood leukocytes, and have not evaluated cells derived from ocular tissue. In this study we evaluated mitochondrial genome variation and heteroplasmy using massively parallel sequencing of mtDNA in a cohort of people with POAG, and in a subset assess the role of somatic mitochondrial genome mutations in disease pathogenesis using paired samples of peripheral blood leukocytes and ocular tissue (Tenon’s ocular fibroblasts).

Herein, we report a novel approach to evaluate somatic mitochondrial genome variants using a paired analysis approach. Pathogenic somatic mitochondrial genome mutations and pathogenic tRNA mutations were observed in people with POAG compared to unaffected people. This supports the evidence of somatic mitochondrial genome variants in the etiology of glaucoma.

## Results

Primary Tenon’s ocular fibroblasts (TFs) were cultured from a subset of POAG patients (GTFs; *n* = 40) and disease negative non-glaucomatous participants (NTFs; *n* = 19). The mean age of the POAG patients from whom TFs were cultured (GTFs) was 70 years (range = 48 to 84) and the mean age of the NTFs patients was 75 years (range 62–89). Blood samples were taken from a larger cohort of POAG patients (*n* = 123; 64 female/59 male) and disease negative (*n* = 95; 61 female/34 male). The mean ages (± standard deviation/SD) of the POAG group were 73.62 (SD ± 10.56) years and the glaucoma negative group were 76.01 (SD ± 8.98) years.

### Mitochondrial genome sequencing of mtDNA from blood leukocytes

The mean coverage for the mitochondrial genome sequencing on mtDNA extracted from blood leukocytes was 2238.0 (± 2140.0) for the POAG participants and 2313.1 (± 1948.9) for the disease negative participants (Supplemental Table 1). A total of 4403 mitochondrial genome variants in 123 POAG participants and 3695 mitochondrial genome variants in 95 disease negative participants were identified (Table 1). The majority of the mitochondrial genome variants in POAG and disease negative participants were heteroplasmic (71% and 75% respectively). The number of homoplasmic variants were significantly higher in POAG participants compared to disease negative (*X*^2^ = 10.21, *p* = 0.001). A higher contribution of known variants were observed in the POAG participants (*X*^2^ = 5.64, *p* = 0.0175). There was no difference observed between the number of transitions and transversions within both groups (*X*^2^ = 0.08, *p* = 0.78).

Variants were classified as pathogenic based on the APOGEE metapredictor classification^49^. There was no significant difference between the mean number of total non-synonymous pathogenic variants per participant in blood-derived mtDNA from control participants (1.48 ± 1.29) compared to POAG participants (1.11 ± 1.18), as confirmed using Mann-Whitney U testing with Bonferroni correction. Similarly, there were no significant differences observed in the mean number of novel non-synonymous pathogenic variants per participant blood-derived mtDNA from disease negative participants (0.19 ± 0.47) compared to POAG participants (0.16 ± 0.39) (*p* = 0.89). A total of 18 pathogenic homoplasmic single nucleotide variants were observed in 13 POAG participants, compared to 11 pathogenic homoplasmic variants in 8 participants in the disease negative group (11% vs. 8%, *X*^2^ = 0.33, *p* = 0.60). The main haplogroups observed were H1, H (other than H1), J and U, which are European haplogroups (Supplemental Table 2) and there was no significant difference in mitochondrial haplotype distribution in POAG compared to disease negative in mtDNA from blood leukocytes. There were also no significant differences in the regional distribution of mitochondrial genome variants (Supplemental Table 3) with the largest number of variants seen in Complex I (29% POAG and 28% disease negative) and the non-coding region (28% POAG and 27% disease negative) reflecting the larger sizes of these regions. 196 tRNA variants were observed in POAG (4% of all variants in POAG: 179 known, 17 novel) compared to 187 tRNA variants in disease negative (5% of all variants in disease negative: 158 known, 13 novel) in mtDNA from blood leukocytes, but these were not significantly different between the two groups. (Supplemental Table 4). Only 5 of these variants were predicted as likely pathogenic using MitoTIP all of which were in POAG.

### Mitochondrial genome sequencing of mtDNA from Tenon’s ocular fibroblasts

The mean coverage for the mitochondrial genome sequencing on mtDNA extracted from Tenon’s ocular fibroblasts (TFs) was 1692.9 (± 1228.7) for the POAG participants (GTFs) and 1412.7(± 1047.0) for the disease negative participants (NTFs). A total of 1818 mitochondrial genome variants in 40 POAG Tenon’s fibroblasts (GTFs) and 812 mitochondrial genome variants in 19 disease negative Tenon’s fibroblasts (NTFs) were identified (Table 1). The majority of the mitochondrial genome variants were heteroplasmic in GTFs (80%) and NTFs (78%). There were no significant differences between the number of novel variants (11% vs. 12%, *X*^2^ = 1.5283, *p* = 0.22), number of transitions/transversions (*X*^2^ = 2.4214, *p* = 0.12) and the number of homoplasmic/heteroplasmic variants (*X*^2^ = 1.07, *p* = 0.30) between the GTFs and NTFs.

**Table 1:**
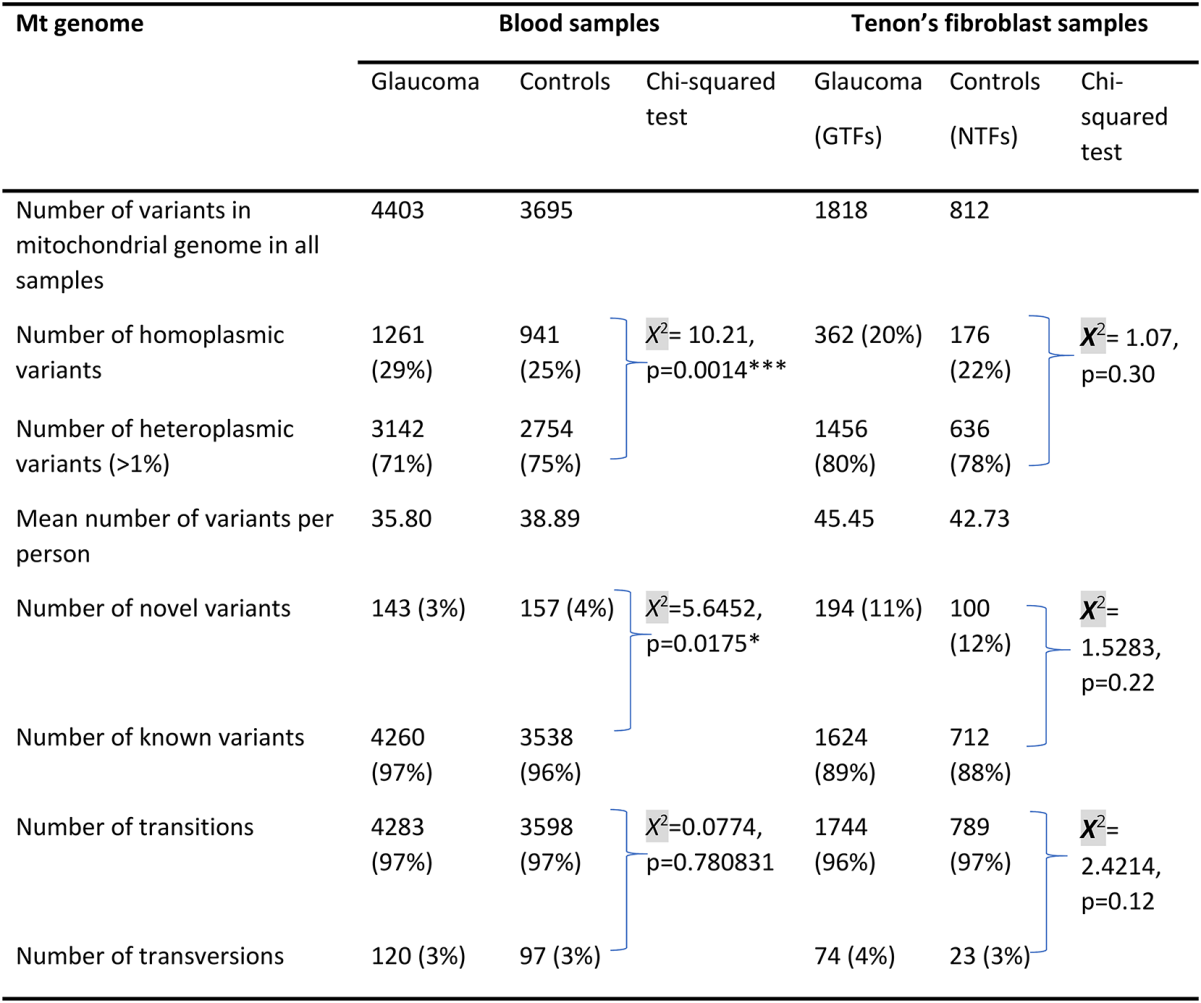
Overview of the number and types of mtDNA variants identified from mitochondrial genome sequencing in POAG and control subjects.

Chi squared testing was used to compare heteroplasmic cf. homoplastic variants, novel cf. known variants, transitions cf. transversions. GTFS- Glaucoma Tenon’s fibroblasts. NTFs- disease negative non-glaucomatous controls Tenon’s fibroblasts *p < 0.05, **p < 0.01, ***p < 0.005, ****p < 0.0001.

Variant classification with the APOGEE metapredictor showed no significant differences between the mean number of total non-synonymous pathogenic variants per participant in GTFs (2.63 ± 1.74) versus NTFs (2.32 ± 1.64) (*p* = 0.55). No significant differences were observed in the mean number of novel non-synonymous pathogenic variants per participant GTFs (0.83 ± 0.93) versus NTFs (0.89 ± 0.88) (*p* = 0.65). A total of 7 known pathogenic homoplasmic variants were demonstrated in 38 GTF samples whilst no pathogenic homoplasmic variants were identified in NTF samples (*n* = 19) (*X*^2^ = 3.92, *p* = 0.0.5). These homoplasmic mtDNA variants were all in complex-I genes: m.13708G > A (MT-ND5, Ala458Thr), 3394T > C I (MT-ND1, Tyr30His) and m.4216T > C (MT-ND1, Tyr304His). The m.13708G > A (MT-ND5, Ala458Thr) variant was detected in 5 GTF samples.

The main haplogroups observed in the TFs were H1, H (other than H1), J, T and U, which are European haplogroups (see Supplemental Table 5) and there were no significant differences between GTFs and NTFs. Mirroring the mtDNA from blood leukocytes there were also no significant differences in the regional distribution of mitochondrial genome variants (Supplemental Table 6) with the largest number of variants seen in Complex I (30% for both NTFs and GTFs) and the non-coding region (26% GTFs and 22% NTFs). 70 tRNA variants (60 known and 10 novel) were observed in GTFs compared to 39 (32 known and 7 novel) in NTFs but these were not significantly different between the two groups. (See Supplemental Table 7). MitoTIP predicted a total of 7 likely pathogenic tRNA variants in GTFs and included four novel variants (m.1669G > A (MT-TV), m.4269 A > C (MT-TI), m.4429G > A (MT-TM) and m.5691G > A (MT-TN) and two known tRNA variants at higher levels of heteroplasmy (m.5522G > A (MT-TW) and m.5628T > C (MT-TA), seen in two samples at a level of 97.21% and 1.03%). In NTF samples, one confirmed known pathogenic variant was identified (m.3243 A > G, MT-TL1) in one participant with a heteroplasmy level of 2.56%. One variant in NTF was predicted as likely pathogenic using MitoTIP (novel variant m.604 A > G, MT-TF, 4.36% heteroplasmy level).

### Paired analysis of mtDNA extracted from Tenon’s ocular fibroblasts and blood

**Fig. 1:**
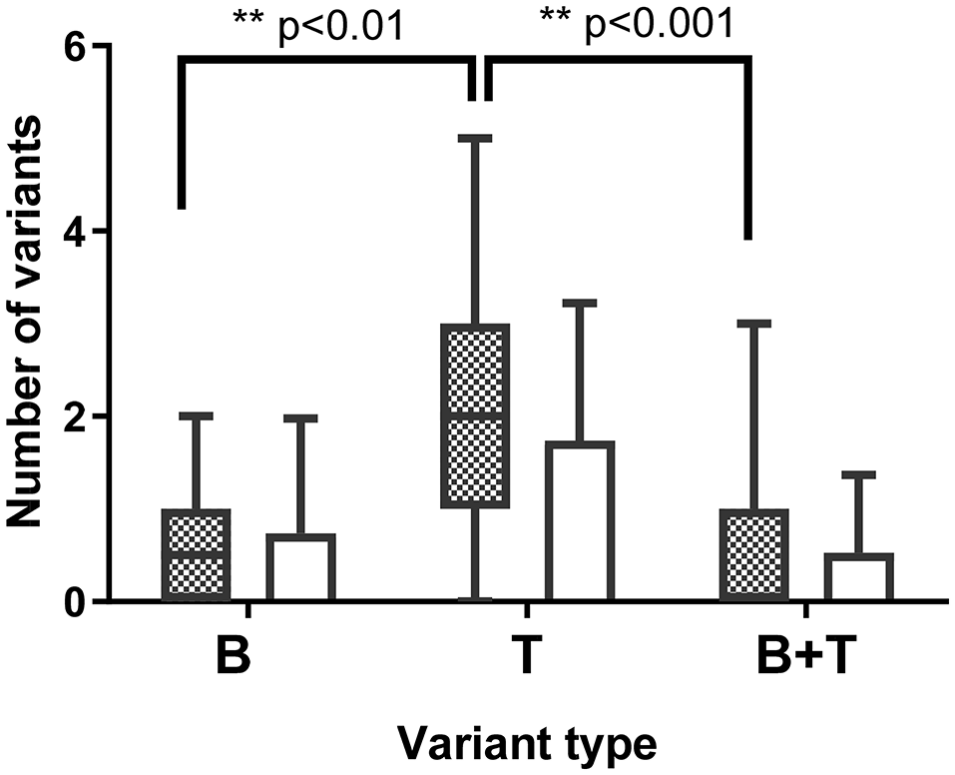
Graphs to show the number of pathogenic somatic Tenon’s fibroblasts variants, germline variants and blood only mtDNA (known and novel) variants in glaucoma and controls. APOGEE metapredictor was used to determine pathogenic variants. Somatic Tenon’s fibroblasts variants are observed in human Tenon’s fibroblasts only but not found in blood samples of each subject analyzed, blood only variants are observed in blood alone but not found in Tenon’s fibroblasts and germline variants are observed in both Tenon’s fibroblasts and blood of each subject analyzed. *p < 0.05, **= *p* < 0.01, ***= *p* < 0.005, **** = *p* < 0.0001. B- Blood only mtDNA variants, T = Tenon’s ocular fibroblasts only variants (somatic mtDNA variants), B + T = Inherited germline mtDNA variants (identical blood and Tenon’s ocular fibroblasts). Shaded- glaucoma, non-shaded- controls.

The paired analysis of mitochondrial genome from Tenon’s ocular fibroblasts and blood from the same participants was a unique aspect of this study. There was a significant increase in somatic nonsynonymous pathogenic variants (Tenon’s ocular fibroblasts only) compared to germline POAG variants samples alone (*p* < 0.001) which was not seen in disease negative (Fig. 1). The number of pathogenic non-synonymous mtDNA variants using the APOGEE metapredictor were characterized as those (1) solely found in the blood, (2) found in both the blood and TFs (germline) and (3) solely found in the TFs (somatic mtDNA variant) of the same participants (Fig. 1). All of the somatic pathogenic Tenon’s ocular fibroblast mtDNA variants were heteroplasmic and not homoplasmic. There were no differences of transitions or transversions between the two groups. Similarly, no differences were observed between the mitochondrial gene region distributions, including tRNA, of pathogenic nonsynonymous somatic Tenon’s variants in POAG and disease negative groups (Table 1). The variants classified as somatic pathogenic mitochondrial genome mutations in NTFs and GTFs with published reports are highlighted in Table 2. There were 16 mitochondrial genome somatic pathogenic variants in POAG participants and 9 in non-glaucomatous participants, which were reported to be associated with disease in MITOMAP.

Novel somatic mitochondrial genome single nucleotide variants were found in GTF and NTF. The novel non-synonymous somatic GTF pathogenic variant m.8090G > A (MT-COII, Gly169Ser) was observed in Tenon’s fibroblasts from two POAG participants: (heteroplasmy levels 18.94% and 1.48%). This variant occurs in the gene for COII, a gene required for complex IV within the mitochondrial respiratory chain and was not observed in the disease negative cohort. These variants were not observed in the corresponding blood mitochondrial genome samples and therefore are deemed pathogenic somatic Tenon’s ocular fibroblast variants. The novel non-synonymous pathogenic somatic Tenon’s ocular fibroblast variants at high levels of heteroplasmy in the POAG participants (m.7662 A > G (MT-COII, His26Arg) heteroplasmy level 75.03%) and m.3413G > C (MT-ND1, Gly36Ala, heteroplasmy level 38.8%)) were not observed in the corresponding paired blood leukocytes, and therefore are somatic Tenon’s ocular fibroblast pathogenic mtDNA variants.

**Table 2:** A table of somatic pathogenic mitochondrial DNA variants reported to be associated with disease in the MITOMAP database, as identified in Tenon’s fibroblasts from glaucoma and control subjects somatic Tenon’s fibroblasts variants are not found in blood samples of each subject analyzed. Pathogenic variants were determined with the APOGEE metapredictor bioinformatic tool. The count refers to the number of times the variant was observed in each cohort. Reference grading score: a grade of 1 had 1 previous report in the literature, 2 had 2 previous reports in the literature and 3 had 3 or more previous reports in the literature. Count represents number of occurrences in glaucoma/control individuals. MELAS- mitochondrial encephalomyopathy, lactic acidosis and stroke like episodes. LHON- Leber’s hereditary optic neuropathy. CPT- carnitine palmitoyltransferase. AMD- age related macula degeneration. NRTI-PN- nucleoside reverse transcriptase inhibitors peripheral neuropathy. LDYT- Leber hereditary optic neuropathy and dystonia. PD- parkinsons disease. HCM- hypertrophic cardiomyopathy. ADS- acquired demyelinating syndrome. MIDD- maternally inherited diabetes and deafness. CPEO- chronic progressive external ophthalmoplegia.

mtDNA variant	Gene	Disease report (MITOMAP)	Reference grading	Glaucoma Count	Control count
m.608 A > G	tRNA F	Tubulo-interstitial nephritis	2	0	1
m.1642G > A	tRNA V	MELAS	2	1	0
m.3380G > A	MT-ND1	MELAS	3	2	0
m.3394T > C	MT-ND1	LHON/Diabetes/CPT deficiency/high altitude adaptation	3	2	0
m.3733G > A	MT-ND1	LHON	3	1	0
m.4136 A > G	MT-ND1	LHON	3	1	0
m.4142G > A	MT-ND1	Development delay, seizure, hypotonia	2	1	0
m.4216T > C	MT-ND1	LHON/Insulin Resistance /possible adaptive high altitude variant	3	5	2
m.4917 A > G	MT-ND2	LHON/Insulin Resistance/AMD/NRTI-PN	3	1	4
m.5244G > A	MT-ND2	LHON	3	0	1
m.5522G > A	tRNA W	Mitochondrial myopathy	1	1	0
m.5628T > C	tRNA A	CPEO/DEAF enhancer/gout	3	1	0
m.6261G > A	MT-COI	Prostate Cancer/LHON	3	1	0
m.9804G > A	MT-COIII	LHON	3	1	0
m.10191T > C	MT-ND3	Leigh Disease/Leigh-like Disease	3	0	1
m.10197G > A	MT-ND3	Leigh Disease/Dystonia/Stroke/LDYT	3	0	1
m.10437G > A	tRNA R	Mitochondrial myopathy	3	1	0
m.12,770 A > G	MT-ND5	MELAS	3	0	1
m.13042G > A	MT-ND5	Optic neuropathy/retinopathy/LD	3	1	0
m.13063G > A	MT-ND5	Adult onset encephalopathy/Ataxia	3	1	0
m.13708G > A	MT-ND5	LHON/Increased MS risk/higher freq in PD-ADS	3	5	0
m.15243G > A	MT-CYB	HCM	3	0	1
m.15915G > A	tRNA T	Encephalomyopathy	2	0	1

### Alterations in mitochondrial copy number and oxidative DNA damage

**Fig. 2:**
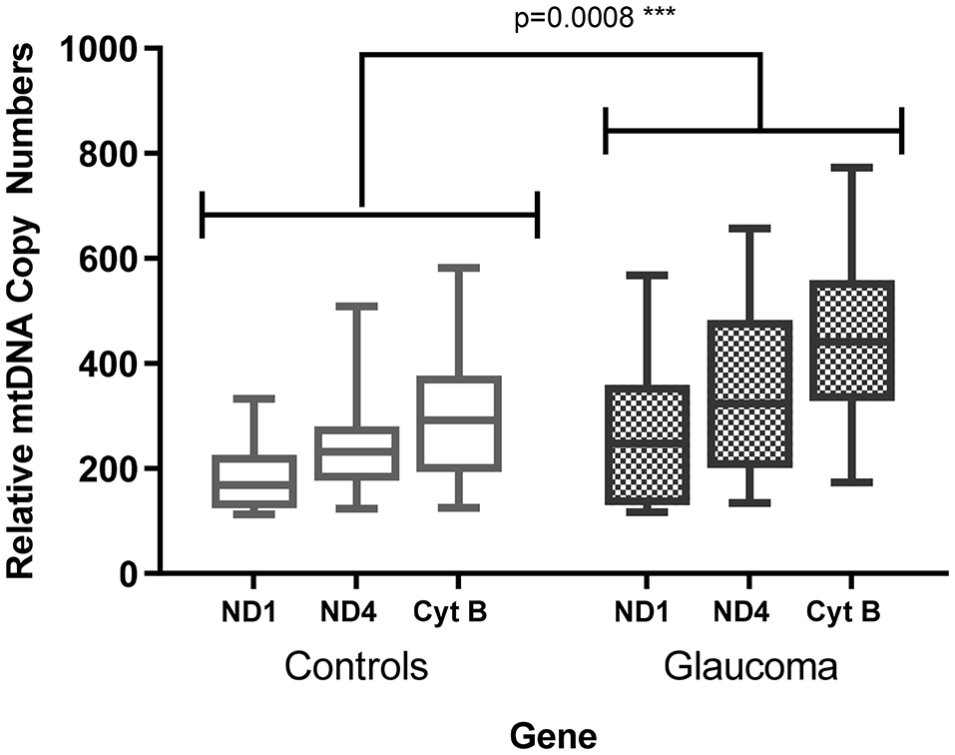
A graph to show the relative mtDNA copy number of three mitochondrial genes (Cyt-B, ND1 and ND4) with normalization to a nuclear gene (PK) after qPCR testing of DNA extracted from glaucoma and control Tenon’s fibroblasts. The mitochondrial genes include ND-1: NADH dehydrogenase gene 1, ND-4: NADH dehydrogenase gene 4, Cyt-B: Cytochrome-B. These were normalized to a nuclear gene (PK: Pyruvate kinase). Two-way ANOVA testing demonstrated a significant increase in glaucoma compared to controls (*p* = 0.0008). qPCR- quantitative polymerase chain reaction. Legend-shaded—glaucoma, non-shaded- controls.

Quantitative PCR was performed on DNA from NTFs (*n* = 15) and GTFs (*n* = 20) (Fig. 2). There was a significant increase in mitochondrial DNA copy number in POAG when compared to controls with two-way ANOVA testing (*p* = 0.0008). There was no significant difference in oxidative DNA damage as measured using 8-OH-dG between GTFs (*n* = 10) and NTFs (*n* = 5) (Supplemental Fig. 1).

## Discussion

Accumulating evidence suggests that mitochondrial dysfunction predisposes neuronal cells to death in age-related neurodegenerative disease such as glaucoma^9,11–14^. In glaucoma, the primary site of pathological changes is the retinal ganglion cells (RGCs), which are only accessible for study in postmortem studies^43^. Murine studies have identified mitochondrial and metabolic defects in RGCs^14,22,50^ and driven clinical studies investigating the role of nicotinamide to prevent neurodegeneration in glaucoma^21,23,24^. Human studies using RGCs are limited, and Tenon’s ocular fibroblasts have been used as a surrogate to identify mitochondrial dysfunction in glaucoma by our group and others^27,45,51^. Herein, we report an enrichment in potentially pathogenic nonsynonymous mtDNA variants Tenon’s ocular fibroblasts in POAG.

Mutations in mtDNA can arise spontaneously through errors in mtDNA replication or through unrepaired oxidative damage to mtDNA^52–55^. The free radical theory of mtDNA mutation proposed that mtDNA was susceptible to oxidative damage due to its proximity to the ETC and to the lack of protective histones^35,52^. This view extended into a vicious cycle theory whereby mitochondrial DNA mutations would result in dysfunctional OXPHOS generating further reactive oxygen species (ROS) inducing further mtDNA mutations and further cell impairment^56^. Basal oxidative stress is elevated in glaucomatous ocular fibroblasts and is not of mitochondrial origin^27^. Oxidative damage to mtDNA usually results in G: C and T: A transversion mutations^52,57^ and the majority of mtDNA variants in our data were transitions. Furthermore, 8-hydroxy-2′-deoxyguanosine (8-OH-dG) a fundamental molecular biomarker of oxidative DNA damage was similar in the POAG and control Tenon’s ocular fibroblasts. The absence of oxidative DNA damage and predominance of transition variants support the concept that errors in mtDNA replication represent the predominant mutation mechanism in glaucomatous ocular fibroblasts. Errors in mtDNA replication are now recognized as the predominant mutation mechanism in mitochondrial genomes^52,57,58^.

Mitochondrial DNA is synthesized during mtDNA replication by low fidelity DNA polymerase γ (pol γ) and spontaneous replication errors by pol γ are responsible for most base substitutions in mtDNA^57^. There was an increased mtDNA copy number in glaucomatous ocular fibroblasts, suggesting increased mtDNA replication as driving somatic mtDNA mutation in POAG^59^. Alterations in mitochondrial DNA copy number are associated with oxidative stress^60^, ageing and disease states: neuromuscular disease, cardiomyopathy, type II diabetes and cancer^61^. The absolute levels of wild type mtDNA are an important determinant of the pathological manifestations of mitochondrial diseases^62^. In LHON, high mtDNA copy number can protect against visual loss^63^. In the glaucomatous ocular fibroblasts, the mtDNA copy number may be protective and a response to defective mitochondrial bioenergetics^27,62^. In fact, in mitochondrial optic neuropathies increasing mtDNA levels has been proposed as a therapeutic strategy which may have relevance to POAG^64^. Interestingly, the mitochondrial transcription factor A (TFAM) is a key regulator of the mitochondrial genome and copy number has been linked to neurodegenerative diseases like Alzheimer’s disease and Parkinson’s disease^65^.

The interpretation of mitochondrial variants is difficult due to the nature of mitochondrial genetics^66^. Determining the most appropriate algorithm to predict the pathogenicity is an important consideration and can impact studies examining mitochondrial genetics^66^. APOGEE was used in this study to determine the pathogenicity of mtDNA non-synonymous variants as it demonstrated higher specificity and sensitivity than other bioinformatics prediction tools^49^. The MITOMAP database was used for the bioinformatics analysis, however, there are a significant proportion of variants that are reported as pathogenic, but not confirmed, and therefore this poses challenges in the molecular diagnosis of mitochondrial diseases^66^. Using GenBank data is also flawed as the sequences may not be of equal quality, some sequences may be present in GenBank with multiple IDs or from multiple cell lines/clones and biased toward data from individuals with pathology^67^. In addition, in normal individuals there is extensive low-frequency high-pathogenic potential mtDNA mutation^68^. Given the potential effects of filtering the mitochondrial genome sequence data using GenBank frequencies or ethnically matched disease negative controls, we used a relatively open agnostic bioinformatics approach. This allowed us to determine and classify potential pathogenic mtDNA variants equally between POAG cases and controls.

Using massively parallel sequencing of the mitochondrial genome and the mtDNA-Server algorithm we were able to detect mtDNA variants at low levels of heteroplasmy (1% and upwards)^37^. The somatic pathogenic nonsynonymous mtDNA variants detected in this study were generally at relatively low levels of heteroplasmy. An important consideration for low levels of heteroplasmy of pathogenic mutations is that they may expand to high frequency levels later in life and hence exceed the phenotypic threshold and result in age related disease^68^. The mechanisms of somatic mtDNA mutation enrichment and clonal expansion are not fully understood^69,70^. Our previous study assessed mtDNA defects in peripheral blood leukocytes but did not specifically address levels of heteroplasmy^28^. In this study we did not detect significant enrichment of mtDNA variants in peripheral blood leukocytes from POAG cases and controls. This finding has been replicated by other massively parallel sequencing studies in POAG^29,30,33^. Mitochondrial DNA studies in blood leukocytes do not replicate the findings in ocular disease. Cells derived from ocular tissues better represent the glaucomatous disease context and can be used as a surrogate to model potential pathogenic mechanisms involved in RGCs degeneration in POAG and therapeutic interventions^27,45,48,51^.

## Materials and methods

### Subjects recruitment and sample collection

Participants with primary open angle glaucoma (POAG) and disease negative non-glaucomatous controls were recruited from St. Paul’s Eye Unit, Royal Liverpool University Hospital. This study adhered to the tenets of Declaration of Helsinki and were approved by the relevant institutions, with all participants giving informed written consent. Ethical approval for the study was acquired from the NHS Research Ethics Committee (REC Ref 14/LO/1088).

Clinical phenotyping included a detailed ocular and medical history, drug history, intraocular pressure (IOP) measurement by Goldmann tonometry, slit-lamp bio-microscopy with stereoscopic disc examination and gonioscopy, and visual field testing (Humprey Visual Field Analyzer, Zeiss; Swedish interactive algorithm standard 24—2 program). The diagnosis of POAG was based on open anterior chamber angles on gonioscopy, glaucomatous optic nerve damage on fundoscopy and a glaucomatous visual field defect. Ethnically matched and age matched controls without glaucomatous optic neuropathy and a pressure less than 21 mmHg, were also recruited to the study. Patients were excluded if below 18 years of age, if they had previous intraocular surgery or any findings on examination suggesting ocular hypertension or a secondary cause of glaucoma. (e.g., pigment dispersion, pseudoexfoliative material in the anterior chamber, uveitis or ocular trauma).

### Isolation of human primary Tenon’s ocular fibroblasts

Human primary Tenon’s ocular fibroblasts (TFs) were cultured from subjects with POAG (GTFs) or non-glaucomatous controls (NTFs) undergoing glaucoma or cataract surgery using the explant method as previously described^47^. A limbal incision was created as a part of glaucoma surgery (or at the site of sub-Tenon’s injection of local anesthetic after administration of topical anesthetic for cataract surgery) and a 5 mm × 5 mm square of Tenon’s tissue was excised from beneath the conjunctiva after separation by blunt dissection. Petri dishes were scored with blade with a middle ‘X’ and the Tenon’s tissue explant was mechanically applied into this central ‘X’. TFs were cultured in complete medium (Dulbecco’s Modified Eagle’s Medium/Nutrient Ham F12 (1:1) medium: DMEM/F12) supplemented with L-glutamine, 10% fetal calf serum, penicillin/streptomycin mix (1:1) and amphotericin (all from Sigma-Aldrich, UK). 5 ml of complete medium was applied and incubated at 37 °C with 5% CO2 and 95% humidity in an incubator (Sanyo CO2 Incubator MCO-17 A, Sanyo, Japan) and cells were passaged until they reached passage 4. The cells were tested for mycoplasma using previously described techniques^71^ and then used for further experiments or conserved at − 80 °C using 10% dimethyl sulfoxide (DMSO) (Sigma-Aldrich, UK) until further use. Vimentin (V9) mouse monoclonal antibody (MA5 11,883) (Thermofisher Scientific, USA) immunocytochemical staining (2 µg/ml in 1% BSA for 1 h at 37 °C) was performed to confirm that the cells were fibroblasts.

### DNA extraction and long-range polymerase chain reaction (LR-PCR) amplification of mitochondrial genome

Genomic DNA was isolated from human peripheral blood leukocytes using the Wizard Genomic DNA Purification kit (Promega, USA) following manufacturer’s instructions. DNA was extracted from TFs at passage 4–6 using QIAGEN DNA extraction kits (AllPrep DNA/RNA/miRNA Universal Kit or DNeasy Blood and Tissue Kit; QIAGEN, Germany). Genomic DNA was isolated following manufacturer’s instructions. The purity and concentration of isolated DNA was determined spectrophotometrically by measuring the absorbance at 260 nm and 280 nm (NanoDrop Technologies; Abtech International UK). The entire human mitochondrial genome was amplified in two overlapping fragments^72^: fragment 1 (5′-AACCAAACCCCAAAGACACC-3′ and 5′-GCC AATAATGACGTGAAGTCC-3′; product size: 9,289 bp) and fragment 2 (5′-TCCCACTCCTAAACACATCC-3′ and 5′-TTT ATGGGGTGATGTGAGCC-3′; product size: 7,626 bp). PCR reactions were performed using a Takara long range PCR kit (Takara Bio USA) in a 25 µl volume consisting of 2 µl of 50ng/µl genomic DNA, 1 µl of 10 µM forward primer and 1 µl of 10 µM reverse primer, 0.125 µl of 5units/µl Takara LA Taq, 2.5 µl of 10x LA PCR Buffer II (Mg^2+^), 4 µl of 2.5mM deoxyribonucleotide triphosphate (dNTP) mixture and 14.375 µl of DNase/RNase free water (Sigma-Aldrich^®^ UK). Thermal cycling was performed using a Veriti 96 well Thermal Cycler (Applied Biosystems, USA) with the following conditions were used: initial denaturation at 94 °C for 1 min followed by 35 cycles of denaturation for 30 s at 94 °C, annealing for 1 min (62 °C for the 9 kb fragment, 64 °C for the 7 kb fragment), extension for 8 min at 72 °C. A final extension step of 5 min at 72 °C was performed and then the samples were cooled to 4 °C. The PCR product was purified using Agencourt AMPure XP Reagent (Beckman Coulter, UK) at 1.8x sample volume. DNA concentrations were subsequently quantified fluorometrically on a Qubit 2.0 Fluorometer (Life Technologies, USA). DNA samples were quantified using the Qubit dsDNA HS Assay Kit (Life Technologies, USA). The PCR products were sent to the Genomics Core Technology Unit at Queen’s University Belfast for library preparation and sequencing.

### Library preparation and massively parallel sequencing of mitochondrial genome

Nextera DNA library Preparation (Illumina, USA) was performed using the Echo 252 Liquid handler (Labcyte, USA). The Nextera XT DNA Library Preparation Kit (FC-131-1096) and Integrated DNA Technologies Inc (IDT) dual indexing primers, compatible with the Nextera workflow were used according to manufacturer’s protocol. The input DNA (pre diluted to 0.2 ng/µL) was fragmented in a tagmentation reaction, and tagged with adapter sequences at either end by the Nextera transposome in a single step. In a subsequent amplification reaction, the tags were then used for primer annealing. The primers introduce dual indexes and the p5 and p7 sequences, resulting in finished library molecules. The individual libraries were then pooled, cleaned-up in a bead wash step, quantified and diluted to 4 nM. The pooled library was then loaded into the NextSeq 500/550 reagent cartridge and subsequently massively parallel sequencing was performed, according to manufacturer’s instructions, on the Nextseq 500 (Illumina, USA). The Illumina sequencing data was transferred from the Illumina BaseSpace cloud and raw data files in binary base call (BCL) format were downloaded and converted to paired-end FASTQ files using bcl2fastq Conversion Software from Illumina.

### Bioinformatics analysis of sequencing data

The FASTQ paired end files were uploaded to the online mtDNA-server for parallel read alignment, homoplasmic variant annotation, heteroplasmy detection and artefact or contamination identification^37^. This aligned the reads to the revised Cambridge reference sequence (rCRS) reference and provided several quality control (QC) metrics^73^. The mtDNA reads with an alignment Phred score ≤ 30 (99% base call accuracy), base quality < 20 (Phred Score: 99% base call accuracy) and a read length < 25 were excluded^74^. Sites showing coverage < 10 bases per strand were filtered and excluded. Post processing was then performed of the output from mtDNA-Server. Samples with a heteroplasmy < 1% were excluded from further analysis. Published evaluation of this server has demonstrated that it can detect heteroplasmies down to the 1% level with perfect specificity and outperforms existing approaches in sensitivity^37^. The mean coverage per sample was analyzed and samples and variants with less than 40 × coverage depth were excluded for further analysis; this minimum coverage was consistent with similar mitochondrial genome sequencing studies^75,76^. The mtDNA-Server classifies heteroplasmies as type 1 (reliable), type 2 (heteroplasmy in a low complexity region) and type 3 (when the major/minor component are swapped for the forward and reverse strand). For our analysis type 3 heteroplasmies were excluded.

Identified mitochondrial genome single nucleotide variants (SNVs) were compared with publicly accessible databases using the MITOMAP database and MITOMASTER analysis^77^. The output provided information of the mutation type, the locus, the translational effect, the references for previous variants submitted to MITOMASTER and the subject phenotype described for that variant from the literature^77^. SNVs were summarized by subject, gene, locus, synonymous/non-synonymous, and previously unreported SNVs were classified as novel if not reported in the 24,187 human mitochondrial DNA sequences in MITOMAP. The pathological impact of the non-synonymous variants was assessed using the predictor MitImpact 32 3.0.1 and the results of the meta predictor classification engine APOGEE was used to determine pathogenicity^78^. MitoTIP was used to predict the pathogenicity of tRNA variants in both groups^79^. MitoTIP is an online tool which integrates multiple sources of information to provide a prediction for the likelihood that novel single nucleotide variants or deletions in tRNA-encoding sequences would cause disease^79^. This provides a grading of likely benign, possibly benign, possibly pathogenic and likely pathogenic. A paired analysis of all non-synonymous and pathogenic non-synonymous variants from each individual study subject (as determined by APOGEE grade) were examined and classified into mtDNA variants as follows; (i) solely found in the blood, (ii) found in both the blood and TFs (germline) and (iii) solely found in the TFs (somatic mtDNA variant) of the same subjects.

### Measurement of mitochondrial copy number

The mitochondrial DNA copy number was determined in this study using qPCR. Relative mtDNA copy numbers were assessed after Cyt-B, ND1 and ND4 normalization by the nuclear gene PK using previously published primers and conditions^80^. All reactions were performed using the Lightcycler^®^ 96 System (Roche, Switzerland) on the DNA extracted from tenons fibroblasts. A total reaction volume of 10 µl was used containing: 5 µl of Maxima SYBR green/Fluorescein qPCR Master Mix 2x (Thermo Scientific, USA), 0.64 µl of the primer pair (320 nm of each primer- primer pair of a 10 µM concentration (Eurofins Genomics, Germany)), 1.0 µl of DNA (concentration of 20 ng/µl) and 3.36 µl of DNAse/RNAse free water (Sigma, UK). Reactions were performed in triplicate. Relative mtDNA copy number was calculated using the following equation: mtDNA/nDNA = 2 − ΔCt, where ΔCt = Ctmito-Ctnuclear^81^.

### 8-OH-dG DNA damage quantification

DNA extracted from Tenon’s fibroblasts were used for analysis of levels of 8-hydroxy-2′-deoxguanosine (8-OHdG). This was performed using the EpiQuik™ 8-OHdG DNA damage quantification direct kit (Colorimetric) (Epigentek USA) as per manufacturer’s instructions. In brief, the anti-8- OHdG monoclonal antibody and the sample or standard were added to a microtiter plate precoated with 8-OHdG. An enzyme-labeled secondary antibody was used as a detection antibody. 220 ng of DNA was used for the purposes of the investigation. All samples were performed in duplicate and an average was taken. Data analysis was performed to determine normality of the data and one way ANOVA testing using Graphpad InStat version 3.10 software (La Jolla, CA, USA).

### Statistical analysis of sequencing results

The statistical analysis of the data was performed using Graphpad InStat version 3.10 software (La Jolla, CA, USA). Mann Whitney U testing was performed for comparisons between glaucoma and controls. In instances where multiple groups were compared Kruskal Wallis testing was used, followed by Post Hoc Dunn’s multiple comparisons if applicable.

## Conclusion

An increase of potentially pathogenic nonsynonymous mtDNA variants were identified in Tenon’s ocular fibroblasts from primary open angle glaucoma participants (GTFs) using massively parallel sequencing when compared to participants without glaucoma (NTFs). Evaluation of mtDNA oxidative damage did not detect changes in the POAG cohort and there was a predominance of transition mtDNA variants. This favours the mutational mechanism of mtDNA in GTFs to relate to errors in mtDNA replication rather than the free radical theory of mtDNA mutation. Our findings support the role of somatic mitochondrial genome variants in the etiology of glaucoma.

**Websites.**

mtDNA-Server: https://mtdna-server.uibk.ac.at.

MITOMAP database: http://www.mitomap.org.

MITOMASTER analysis: http://mitomap.org/MITOMASTER.

MitImpact 32 3.0.1: http://mitimpact.css-mendel.it/.

## Supplementary Information

Supplementary Material 1.

## Data Availability

Sequence data that support the findings of this study has been uploaded: PRJNA1138252This will be made publically available upon publication.

## Abbreviations

8-OHdG: 8-Hydroxy-2′-deoxguanosine
ADOA: Autosomal dominant optic atrophy
DMEM: Dulbecco’s Modified Eagle’s Medium
DMSO: Dimethyl sulfoxide
ETC: Electron transport chain
GTFs: Glaucomatous Tenon’s fibroblasts
IOP: Intraocular pressure
LHON: Leber’s hereditary optic neuropathy
LR: PCR-long range polymerase chain reaction
mtDNA: Mitochondrial DNA
NTFs non: Glaucomatous controls
OXPHOS: Oxidative phosphorylation
PCR: Polymerase chain reaction
POAG: Primary open angle glaucoma
QC: Quality control
qPCR: Quantitative polymerase chain reaction
rCRS: Revised Cambridge reference, sequence
RGC: Retinal ganglion cells
SD: Standard deviation
SNV: Single nucleotide variants
TFs: Tenon’s fibroblasts
tRNA: transfer RNA
