# Supplementary material for: Massively parallel sequencing of mitochondrial genome in primary open angle glaucoma identifies somatically acquired mitochondrial mutations in ocular tissue
Source: Sci Rep. 2024 Nov 1;14:26324. doi: 10.1038/s41598-024-72684-6 (PMC11530638; doi:10.1038/s41598-024-72684-6)

Supplemental Figures

Supplemental Table 1: A table to show the mtDNA sequencing coverage statistics and the type of heteroplasmies observed in the mtDNA server output from Tenon's fibroblast and blood DNA in glaucoma and control subjects

Coverage is the number of unique reads for each run. Type 2 heteroplasmy = heteroplasmy in low complexity region; Type 3 heteroplasmy= Major/Minor component are swapped for forward and reverse strand. Heteroplasmy is calculated as a percentage by dividing the total number of variants at a certain nucleotide position by the total number of genomes represented at the position multiplied by 100. Samples with a heteroplasmy <1% were excluded from further analysis as the server has demonstrated that it can detect heteroplasmies down to the 1% level with perfect specificity.

	**Glaucoma Tenon's**	**Non Glaucoma Tenon's**	**Glaucoma Blood**	**Non Glaucoma Blood**
Minimum coverage (number of reads)	60.5	100.5	42	42
Maximum coverage (number of reads)	9121	8395.5	20392	31582
Mean Coverage (number of reads)	1692.9	1412.7	2238.0	2313.1
Standard deviation	1228.7	1047.0	2140.035	1948.9
Type 2 heteroplasmy	232	122	330	456
Type 3 heteroplasmy	27	11	27	20
Total number of variants found with less than 5% heteroplasmy	791	292	1346	1281
Total number of variants found with less than 1% heteroplasmy	525	272	749	1129

Supplemental table 2: A table to show the mitochondrial haplogroup composition and comparison in glaucoma and non glaucoma participants from mtDNA sequencing of peripheral blood samples

Odds ratios were used for comparison of the two groups. Statistical significance between the groups could only be observed after Bonferroni correction for multiple comparisons in post hoc mitochondrial haplogroup analysis if $p < 0.005$. Rare haplogroups or those with single or low incidence were grouped together as 'Other' haplogroups. No significant difference was observed between the two groups.

Haplogroup	Glaucoma participants (n= 123)	Non glaucoma participants (n= 95)	P value	Odds ratio (95% confidence interval)
H1	26 (21%)	16 (17%)	0.424	1.3235 (0.6638- 2.6386)
H (other than H1)	27 (22%)	16 (17%)	0.348	1.3887 (0.6991- 2.7584)
I	5 (4%)	2 (2%)	0.473	1.9703 (0.3738- 10.3857)
J	17 (14%)	16 (13%)	0.538	0.7919 (0.3770-1.6634)
K	6 (5%)	9 (9%)	0.183	0.4900 (0.1681- 1.4284)
L	4 (3%)	1 (1%)	0.390	3.1597 (0.3473- 28.7443)
T	9 (7%)	13 (14%)	0.122	0.498 (0.2033- 1.2201)
U	16 (13%)	11 (12%)	0.752	1.142 (0.5034-2.5904)
V	2 (2%)	5 (5%)	0.244	0.2975 (0.0564- 1.5685)
Others (C, D, G, HV, I, N, R, W, X, Z)	11 (9%)	6 (6%)	0.613	1.4568 (0.5186- 4.0927)

Suppl Table 3: A table to show the distribution of all sequence variants observed and the mean number of variants per person in different regions of the mitochondrial genome, as identified by mtDNA sequencing in blood samples from glaucoma and non glaucoma participants

Regions evaluated included the rRNA coding region (MT-RNR1 (12S) and MTRNR2 (16S) genes), the tRNA coding region (for 22tRNAs) and the coding regions for the proteins of the respiratory chain complexes including complex I (MT-ND1-4, MT-ND4L, MT-ND5-6), complex III (MT-CYB), complex IV (MT-CO1-3) and ATP synthase (MT-ATP6 and MT-ATP8), and the non-coding region. The mean number of variants was calculated per person and compared between the two groups and Mann-Whitney U test was used to compare the two groups. Statistical significance after Bonferroni correction for multiple comparisons in post hoc variant analysis is p < 0.00625. SD- standard deviation. No significance observed between the two groups.

Mitochondrial regions	Number of all variants		Mean number of variants per person (Mean ±SD)		Mann-Whitney U test value	P Value
	Glaucoma	Non glaucomas	Glaucoma	Non glaucoma		
Total	4403	3695	35.80±14.66	38.89±19.29	5350.5	0.29
rRNA	632	550	5.14 ±1.97	5.79±2.58	4997.0	0.06
tRNA	196	187	1.59±1.37	1.97±1.93	5451.5	0.39
Complex I	1283	1048	10.43±5.72	11.02±7.17	5653.5	0.68
Complex III	472	409	3.84±2.24	4.31±2.22	5057.0	0.09
Complex IV	340	300	2.76±2.04	3.16±2.39	5347.5	0.28
ATP synthase	249	198	2.02±1.07	2.08±1.25	5817.5	0.96
Non coding	1231	1003	10.08±4.24	10.56±6.03	5794.0	0.92

Suppl Table 4: A table of the MitoTIP grading of mitochondrial tRNA variants and mean number of variants per person observed in glaucoma and non glaucoma blood samples

MitoTIP was used to determine pathogenicity of mitochondrial tRNA variants. **Mann-Whitney U test was used to compare the two groups. Statistical significance after Bonferroni correction for multiple comparisons in post hoc variant analysis is p <0.0083. SD- standard deviation. No significance observed between the two groups.**

MitoTIP grading	Number of all variants		Mean number of variants per person (Mean ±SD)		Mann-Whitney U test value	P Value
	Glaucoma	Non glaucoma	Glaucoma	Non glaucoma		
Total	196	187	1.59±1.37	1.98±1.93	5480.5	0.42
Likely benign	120	110	0.98±1.09	1.16±1.45	5176.0	0.77
Possibly benign	65	68	0.53±0.73	0.75±0.72	4901.0	0.02
Possibly pathogenic	6	9	0.04±0.22	0.09±0.29	5574.0	0.19
Likely pathogenic	5	0	0.04±0.20	0	N/A	N/A
Confirmed pathogenic	0	0	0	0	N/A	N/A

Suppl Table 5: A Table to show the mitochondrial haplogroup composition and comparison of Tenon's fibroblasts from glaucoma and non glaucoma participants

Odds ratios were used for comparison of the two groups. Statistical significance between the groups could only be observed after Bonferroni correction for multiple comparisons in post hoc mitochondrial haplogroup analysis if p < 0.005. Rare haplogroups or those with single or low incidence were grouped together as 'Other' haplogroups. No significant difference was observed between the two groups.

Haplogroup	Glaucoma participants (n= 38)	Non glaucoma participants (n= 19)	P Value	Odds ratio (85% confidence interval)
H1	12 (32%)	1 (5%)	0.042	8.3077 (0.9905- 69.6787)
H (other than H1)	9 (24%)	6 (32%)	0.522	0.06724 (0.198- 2.2838)
I	1 (3%)	1 (5%)	>0.999	0.4865 (0.0288- 8.2314)
J	8 (21%)	2 (11%)	0.469	2.2667 (0.4311- 11.9191)
K	3 (8%)	1 (5%)	>0.999	1.5429 (0.1496-15.9133)
L	1 (3%)	0	N/A	N/A
T	1 (3%)	3 (15%)	0.103	0.1441 (0.0139- 1.4933)
U	2 (5%)	4 (21%)	0.164	0.2083 (0.0344- 1.2618)
V	0	0	N/A	N/A
Others (A, C, D, G, HV, N, R, W, X, Z)	1 (3%)	1 (5%)	>0.999	0.4865 (0.0288- 8.2314)

Suppl Table 6 : A table to show the distribution of all sequence variants observed and the mean number of variants per person in different regions of the mitochondrial genome, as identified by mtDNA sequencing in Tenon's fibroblasts from GTFs and NTFs

Mann-Whitney U test was used to compare the two groups. Statistical significance after Bonferroni correction for multiple comparisons in post hoc variant analysis is $p < 0.00625$. SD- standard deviation. No significance observed between the two groups. GTFS- Tenon's fibroblasts from glaucoma. NTFs- Tenon's fibroblasts from participants without glaucoma

Mitochondrial regions	Number of all variants		Mean number of variants per person (Mean ±SD)		Mann-Whitney U test value	P Value
	Glaucoma (GTFs)	Non glaucoma (NTFs)	Glaucoma (GTFs)	Non glaucoma (NTFs)		
All	1818	812	45.45±17.25	42.74±10.08	367.50	0.85
rRNA	280	138	7.00±2.77	7.26±2.05	334.50	0.46
tRNA	70	39	1.75±1.53	2.05±1.03	312.00	0.26
Complex I	555	246	13.88±6.42	12.95±5.25	349.00	0.62
Complex III	205	80	5.13±2.36	4.21±1.75	312.50	0.27
Complex IV	160	85	4.00±2.95	4.47±1.84	295.00	0.17
ATP synthase	80	43	2.00±0.93	2.26±1.24	343.00	0.53
Non coding	468	181	11.70±4.68	9.53±3.79	286.00	0.13

Suppl Table 7: A table of the MitoTIP grading of mitochondrial tRNA variants and mean number of variants per person observed in GTFs and NTFs

MitoTIP was used to determine pathogenicity of mitochondrial tRNA variants. **Mann-Whitney U test was used to compare the two groups. Statistical significance after Bonferroni correction for multiple comparisons in post hoc variant analysis is p < 0.0083. SD- standard deviation. No significance observed between the two groups.** GTFS- Tenon's fibroblasts from glaucoma. NTFs- Tenon's fibroblasts from participants without glaucoma

MitoTIP grading	Number of all variants		Mean number of variants per person (Mean ±SD)		Mann-Whitney U test value	P Value
	Glaucoma (GTFs)	Non glaucoma (NTFs)	Glaucoma (GTFs)	Non glaucoma (NTFs)		
Total	70	39	1.75± 1.53	2.05± 1.03	312.00	0.26
Likely benign	36	17	0.9± 1.13	0.89± 0.81	353.00	0.65
Possibly benign	23	14	0.58± 0.78	0.74± 0.65	315.00	0.25
Possibly pathogenic	4	6	0.10 ±0.38	0.30± 0.47	313.00	0.03
Likely pathogenic	7	1	0.15± 0.36	0.05± 0.23	343.00	0.29
Confirmed pathogenic	0	1	0	0.05± 0.23	N/A	

Supplemental Figure 1: A graph to show the levels of 8-OHdG DNA damage after testing genomic DNA extracted from Tenon’s fibroblasts, using the EpiQuik™ 8-OHdG quantification direct kit in glaucoma and non glaucoma groups

Data was analysed using unpaired t testing between the two groups and this demonstrated no significant difference in levels of 8-OHdG between glaucoma and Non glaucoma ($p=0.5356$). 8-OHdG-8-hydroxy-2’-deoxguanosine

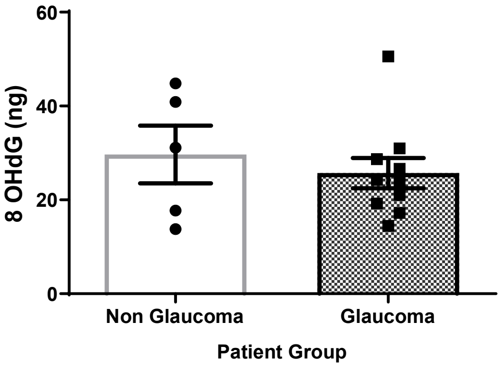

Supplement: Supplementary file 1 — Supplementary Material 1. [file 41598_2024_72684_MOESM1_ESM.pdf]
